# Supplementary material for: Influence of landscape structure on carbon storage in agroforestry systems with cacao and silvopastoral systems in the Colombian Amazon
Source: PLoS One. 2025 Jun 23;20(6):e0325477. doi: 10.1371/journal.pone.0325477 (PMC12184915; doi:10.1371/journal.pone.0325477)
Supplement: Inclusivity in global research questionnaire — (PDF) [file pone.0325477.s001.pdf]

Inclusivity in global research

PLOS' policy on inclusivity in global research aims to improve transparency in the reporting of research performed outside of researchers' own country or community and ensures that PLOS publications reporting global research adhere to high standards for research ethics and authorship. Authors of relevant research articles may be asked to complete the questionnaire below, which outlines ethical, cultural, and scientific considerations specific to inclusivity in global research. This questionnaire may be requested when researchers have travelled to a different country to conduct research, if research uses samples collected in another country, research with Indigenous populations or their lands, or if research is on cultural artefacts. Researchers travelling to another country solely to use laboratory equipment will not normally be required to complete the questionnaire. However, the questionnaire can be requested at the journal's discretion for any submission – if you have been requested to complete this questionnaire by the PLOS journal you submitted to, please do so.

Please complete the questionnaire below and include this as a Supporting Information file with your manuscript. Note that if your paper is accepted for publication, this checklist will be published with your article in the supporting information files. Please ensure that you reference the checklist in the main body of your manuscript. We suggest adding a subsection 'Inclusivity in global research' to your Methods section and adding the following sentence: "Additional information regarding the ethical, cultural, and scientific considerations specific to inclusivity in global research is included in the Supporting Information (SX Checklist)"

The questions have been designed to be applicable to a wide range of study types, and there are subsections for both human subjects research and non-human subjects research. If any of the questions are not relevant to your research please mark them as "N/A" as appropriate.

Ethical considerations, permits and authorship

This section is applicable to all research types.

Provide details as to who granted permissions and/or consent for the study to take place in the Methods section of your manuscript. This should include the names of **all** ethics boards, governmental organizations, community leaders or other bodies that provided approval for the study. If individuals provided approval refer to these people by their role or title but do not list their name(s).

Reported on page number: The research was conducted under the framework permit for the collection of wild biological diversity specimens for non-commercial scientific research purposes. This permit was granted by the Corporation for the Sustainable Development of the Southern Amazon – CORPOAMAZONIA to the University of the Amazon and its Research Groups, through Resolution 1006 of 2014.

If there were any deviations from the study protocol after approval was obtained please provide details of these changes in the Methods section of your manuscript.

Reported on page number: There were no deviations from the study protocol after approval was obtained for the implementation of the methods within the research.

Did this study involve local collaborators that are residents of the country where the research was conducted or members of the community studied? If you do not have any authors from said communities, please provide an explanation for this below.

Yes, the study involved members of local rural communities. These individuals were the owners of the cattle and cacao farms where data collection activities were carried out as part of the research. These communities are part of second-level associations, through which contact was established and access permissions were obtained. The associations are the Comité Departamental de Ganaderos del Caquetá (Departmental Cattle Ranchers Committee of Caquetá) and the Asociación Departamental de Cultivadores de Cacao y Especies Maderables del Caquetá – ACAMAFRUT (Departmental Association of Cacao and Timber Species Growers of Caquetá).

Everyone listed as an author should meet PLOS' criteria for authorship and all individuals who meet these criteria should be included in the author byline, rather than the acknowledgements. For further information please see the journal's Authorship Policy.

Human subjects research (e.g. health research, medical research, cross-cultural psychology)

Did you obtain written informed consent from a representative of the local community or region before the research took place? How did you establish who speaks for the community? Details of written informed consent obtained from study participants should be reported separately in the Methods section of your manuscript.

This research did not involve any human subjects.

How did members of the local community provide input on the aims of the research investigation, its methodology, and its anticipated outcome(s)?

The research did not include the participation of communities within the methodology or data collection. The collaboration process was aimed at obtaining access permits for the cattle and cacao farms. This collaboration was established between the Andean Amazon Biodiversity Research Center (INBIANAM) of the University of the Amazon and the productive sector associations, who agreed to collaborate in providing access to the sites for collecting dendrometric data of trees within the carbon plots.

When engaging with the local community, how did you ensure that the informed consent documents and other materials could be understood by local stakeholders?

Considering that this research did not involve human subjects, the only interaction was the access permission to the farms. This was achieved through the dissemination of the research characteristics to the professionals and technicians of the two guild organizations that advise the producers. They acted as mediators in the process of obtaining access permits for the establishment and census of the carbon plots.

Will the findings of the research be made available in an understandable format to stakeholders in the community where the study was conducted (e.g. via a presentation, summary report, copies of publications, etc.)? Please provide details of how this will be achieved.

Yes, in fact, meetings and courses have already been held where biodiversity and ecosystem services of cacao agroforestry systems and silvopastoral systems in the Colombian Amazon are shared. Additionally, a package of raw data obtained from carbon measurements was provided to the ACAMAFRUT association to be included in a project proposal for the benefit of producers within the framework of economic incentives for conservation.

Non-human subjects research using specimens/ animals collected as part of the study, or those housed in archival collections. Examples include archaeology, paleontology, botany and zoology.

Did the permission you obtained from a local authority to perform the study include an agreement on access to outputs and benefit sharing? This may include procedures to enable fair distribution of the benefits and resources arising from the research performed. Please include any details of Prior Informed Consent and Benefit Sharing Agreements obtained. These may be required by field-specific regulations, for example the Convention on Biological Diversity (CBD) and the associated Nagoya Protocol.

The research was conducted under the framework permit for the collection of wild biological diversity specimens for non-commercial scientific research purposes. This permit was granted by the Corporation for the Sustainable Development of the Southern Amazon – CORPOAMAZONIA to the University of the Amazon and its Research Groups, through Resolution 1006 of 2014. Additionally, the information collected in the field was made available to the two guilds and communities where the research took place.

If the material used in your study was imported, please A) provide the year it was imported and B) indicate whether permits were obtained to import/export the materials used, C) provide details of any permits obtained. If this information is not available, please indicate this.

No material used in the research was imported or exported.

If you used archival specimens, please state how the material used in your study was acquired by the institute it is held in and provide details of any permits obtained for the original excavations/ sample collection. If this information is not available, please indicate this.

In this research, no archival specimens were used; the data utilized stem from measurements of dendrometric variables of trees and shrubs within the plots, taken directly in the field.

How was the potential cultural significance of the materials collected in your study to local communities considered in your research design? Were Indigenous peoples and/or local researchers and institutions involved with archaeological excavations / collection of specimens? If so, please provide a description of their involvement.

In this research, no archaeological excavations or specimen collections were conducted; only in situ measurements of trees and shrubs found within the plots for carbon measurement in the productive systems were carried out.

If your manuscript includes photographs of human remains please indicate whether authors obtained permission from descendants or affiliated cultural communities to do so.

The manuscript does not include photographs of human remains.
