# Supplementary material for: Influence of landscape structure on carbon storage in agroforestry systems with cacao and silvopastoral systems in the Colombian Amazon
Source: PLoS One. 2025 Jun 23;20(6):e0325477. doi: 10.1371/journal.pone.0325477 (PMC12184915; doi:10.1371/journal.pone.0325477)
Supplement: S1–S4 Tables — (DOCX) [file pone.0325477.s002.docx]

**S1 Table. Mean values of landscape metrics by class in the vegetation covers classified in the thematic map in landscape mosaics with cocoa agroforestry systems (SAFc) and silvopastoral systems (SSP) of the Colombian Amazon.**

| **System** | **Mosaics** | **VC** | **Class** | **Landscape metrics** | | | | | | |
| --- | --- | --- | --- | --- | --- | --- | --- | --- | --- | --- |
|  |  |  |  | **CA** | **AREA** | **GYRATE** | **SHAPE** | **FRAC** | **PARA** | **CONTIG** |
| SAFc | BA | CAC | BACAC | 8.59 | 4.30 | 87.49 | 1.46 | 1.07 | 295.00 | 0.97 |
|  |  | PAD | BAPAD | 15.39 | 15.39 | 178.95 | 2.54 | 1.16 | 260.42 | 0.97 |
|  |  | RTT | BARTT | 11.22 | 2.80 | 88.69 | 2.21 | 1.14 | 535.10 | 0.94 |
|  |  | RTV | BARTV | 23.64 | 23.64 | 206.76 | 1.89 | 1.10 | 156.32 | 0.98 |
|  | SR | BSE | SRBSE | 1.97 | 1.97 | 65.55 | 1.69 | 1.11 | 482.85 | 0.93 |
|  |  | CAC | SRCAC | 5.18 | 2.59 | 80.33 | 1.58 | 1.09 | 403.27 | 0.94 |
|  |  | RTT | SRRTT | 14.80 | 3.70 | 74.04 | 1.82 | 1.12 | 694.50 | 0.89 |
|  |  | RTV | SRRTV | 17.21 | 8.61 | 169.84 | 2.56 | 1.14 | 443.54 | 0.93 |
|  | TE | CAC | TECAC | 6.93 | 3.47 | 73.80 | 1.29 | 1.05 | 280.49 | 0.96 |
|  |  | RTT | TERTT | 56.66 | 18.89 | 140.99 | 1.56 | 1.08 | 359.79 | 0.95 |
|  |  | RTV | TERTV | 9.63 | 4.81 | 135.41 | 2.13 | 1.14 | 393.38 | 0.94 |
|  | TR | CAC | TRCAC | 10.83 | 10.83 | 126.96 | 1.38 | 1.06 | 168.64 | 0.98 |
|  |  | PAD | TRPAD | 24.50 | 24.50 | 202.91 | 1.98 | 1.12 | 246.09 | 0.97 |
|  |  | RTT | TRRTT | 5.99 | 1.20 | 51.17 | 1.67 | 1.12 | 962.83 | 0.88 |
| SSP | ES | PAD | ESPAD | 30.83 | 8.87 | 137.81 | 2.03 | 1.13 | 374.90 | 0.94 |
|  |  | RTV | ESRTV | 20.71 | 10.35 | 157.72 | 2.29 | 1.15 | 366.50 | 0.94 |
|  | PO | PAD | POPAD | 12.33 | 12.33 | 169.79 | 2.47 | 1.16 | 401.73 | 0.95 |
|  |  | RTT | PORTT | 15.87 | 15.87 | 301.65 | 3.43 | 1.21 | 346.13 | 0.95 |
|  |  | RTV | PORTV | 11.95 | 5.97 | 110.50 | 1.47 | 1.07 | 243.97 | 0.97 |
|  | VE | BSE | VEBSE | 8.14 | 4.07 | 87.89 | 1.42 | 1.07 | 304.12 | 0.95 |
|  |  | RTT | VERTT | 44.70 | 44.70 | 304.67 | 2.36 | 1.13 | 141.34 | 0.98 |
|  |  | RTV | VERTV | 28.19 | 5.64 | 102.98 | 1.59 | 1.08 | 1727.25 | 0.77 |
|  | VM | BSE | VMBSE | 17.78 | 17.78 | 183.31 | 1.43 | 1.06 | 135.44 | 0.98 |
|  |  | PAD | VMPAD | 27.13 | 12.06 | 139.30 | 1.65 | 1.09 | 336.78 | 0.96 |
|  |  | RTT | VMRTT | 26.60 | 6.65 | 100.04 | 1.85 | 1.12 | 571.29 | 0.94 |

VC: Vegetation covers; SR: Santa Rosa; VE: La Vega ; PO: El Porvenir; BA: Batalla 13; VM: Villa Mery; TR: El Triunfo; ES: Esmeraldas; TE: El Tesoro; BSE: Secondary forest; CAC: Agroforestry cacao cultivation; PAD: Pastures with scattered trees; RTT: Early Fallows; RTV: Old Fallows.

**S2 Table. Matrix of dasometric variables taken in the temporary plots installed in the eight landscape mosaics with agroforestry systems with cocoa and silvopastoral systems in the Colombian Amazon**

| **System** | **Locality** | **Mosaics** | **Parcel** | **Parcel code** | **VC** | **Age** | **Abundance individuals** | | | | | | **DBH** | **Height** | **AGB** |
| --- | --- | --- | --- | --- | --- | --- | --- | --- | --- | --- | --- | --- | --- | --- | --- |
|  |  |  |  |  |  |  | **CS** | **HbT** | **CI** | **FT** | **ST** | **PA** |  |  |  |
| SAFc | El Doncello | El Triunfo (TR) | P01 | TRT1P1 | PAD | 18.0 |  |  | 1 |  | 23 |  | 26.65 | 10.17 | 2502.01 |
|  |  |  | P02 | TRT1P2 | CAC | 25.0 | 25 |  |  |  | 13 |  | 22.13 | 7.42 | 2393.94 |
|  |  |  | P03 | TRT2P1 | PAD | 18.0 |  |  |  |  | 12 |  | 14.34 | 8.13 | 692.10 |
|  |  |  | P04 | TRT2P2 | RTT | 6.0 |  |  |  |  | 7 |  | 28.56 | 9.07 | 625.60 |
|  |  |  | P05 | TRT3P1 | RTT | 6.0 |  |  |  |  | 28 | 4 | 26.66 | 10.16 | 3472.93 |
|  |  |  | P06 | TRT3P2 | PAD | 18.0 |  |  | 3 | 1 | 9 |  | 19.55 | 5.46 | 2091.98 |
|  |  |  | P07 | TRT4P1 | CAC | 25.0 | 4 |  | 1 |  | 25 |  | 29.80 | 11.03 | 3064.47 |
|  |  |  | P08 | TRT4P2 | CAC | 25.0 | 16 | 1 | 3 |  | 16 |  | 22.09 | 7.22 | 3377.74 |
|  |  |  | P09 | TRT5P1 | CAC | 25.0 | 6 |  | 3 |  | 16 |  | 23.67 | 9.24 | 2868.97 |
|  |  |  | P10 | TRT5P2 | CAC | 25.0 | 25 |  | 1 |  | 11 |  | 22.55 | 7.18 | 2718.01 |
|  | Florencia | Batalla 13 (BA) | P11 | BAT1P1 | RTV | 6.0 |  |  |  |  | 10 |  | 12.70 | 6.40 | 530.47 |
|  |  |  | P12 | BAT1P2 | RTT | 7.0 |  |  |  |  | 33 |  | 18.22 | 11.25 | 2242.59 |
|  |  |  | P13 | BAT2P1 | RTT | 8.0 |  |  |  |  | 42 |  | 25.67 | 11.56 | 3505.98 |
|  |  |  | P14 | BAT2P2 | RTV | 14.0 |  |  |  |  | 42 |  | 17.82 | 11.19 | 2796.80 |
|  |  |  | P15 | BAT3P1 | RTV | 12.0 |  |  |  |  | 29 |  | 18.96 | 9.72 | 1997.00 |
|  |  |  | P16 | BAT3P2 | CAC | 8.0 | 9 |  |  |  | 15 |  | 29.79 | 11.00 | 1883.42 |
|  |  |  | P17 | BAT4P1 | RTT | 7.5 |  |  |  |  | 33 | 5 | 19.39 | 8.66 | 3820.60 |
|  |  |  | P18 | BAT4P2 | CAC | 8.0 | 43 | 28 |  |  | 1 |  | 15.89 | 5.95 | 7355.69 |
|  |  |  | P19 | BAT5P1 | PAD | 14.0 |  |  |  |  | 17 |  | 21.81 | 10.96 | 1299.43 |
|  |  |  | P20 | BAT5P2 | RTT | 6.8 |  |  |  |  | 28 |  | 19.79 | 7.12 | 1986.63 |
|  | La Montañita | El Tesoro (TE) | P21 | TET1P1 | RTT | 8.5 |  |  |  |  | 19 |  | 12.42 | 3.68 | 992.65 |
|  |  |  | P22 | TET1P2 | RTT | 7.0 |  |  |  |  | 12 |  | 12.63 | 3.71 | 635.55 |
|  |  |  | P23 | TET2P1 | RTT | 8.0 |  |  |  |  | 33 |  | 17.48 | 10.91 | 2150.24 |
|  |  |  | P24 | TET2P2 | RTT | 7.5 |  |  |  |  | 21 |  | 14.70 | 5.71 | 1225.84 |
|  |  |  | P25 | TET3P1 | CAC | 7.5 | 38 | 24 |  |  | 2 |  | 13.52 | 4.26 | 2592.76 |
|  |  |  | P26 | TET3P2 | RTV | 17.0 |  |  |  |  | 56 |  | 18.81 | 9.66 | 3846.94 |
|  |  |  | P27 | TET4P1 | CAC | 6.5 | 41 | 32 |  |  | 4 |  | 13.98 | 4.64 | 3615.24 |
|  |  |  | P28 | TET4P2 | RTT | 8.0 |  |  |  |  | 36 |  | 12.94 | 4.53 | 1936.17 |
|  |  |  | P29 | TET5P1 | RTV | 17.0 |  |  |  |  | 16 |  | 25.74 | 9.94 | 1354.87 |
|  |  |  | P30 | TET5P2 | RTT | 9.0 |  |  |  |  | 38 |  | 14.21 | 5.55 | 2176.96 |
|  | San José del Fragua | Santa Rosa (SR) | P31 | SRT1P1 | RTV | 16.0 |  |  |  |  | 64 | 5 | 17.93 | 12.36 | 4598.92 |
|  |  |  | P32 | SRT1P2 | RTV | 15.0 |  |  |  |  | 35 | 3 | 29.35 | 14.63 | 3453.03 |
|  |  |  | P33 | SRT2P1 | BSE | 25.0 |  |  |  |  | 63 | 8 | 21.73 | 12.46 | 6285.98 |
|  |  |  | P34 | SRT2P2 | CAC | 14.0 | 53 |  |  |  |  |  | 14.77 | 3.50 | 1749.05 |
|  |  |  | P35 | SRT3P1 | RTV | 17.0 |  |  |  |  | 71 |  | 25.18 | 17.14 | 5889.27 |
|  |  |  | P36 | SRT3P2 | CAC | 14.0 | 66 |  |  |  | 3 |  | 12.03 | 3.90 | 1426.34 |
|  |  |  | P37 | SRT4P1 | BSE | 25.0 |  |  |  |  | 65 | 4 | 18.77 | 11.57 | 4707.60 |
|  |  |  | P38 | SRT4P2 | CAC | 9.0 | 4 | 20 |  |  | 3 |  | 15.43 | 4.83 | 2929.54 |
|  |  |  | P39 | SRT5P1 | RTV | 16.5 |  |  |  |  | 47 |  | 22.08 | 13.93 | 3579.21 |
|  |  |  | P40 | SRT5P2 | RTT | 10.0 |  |  |  |  | 30 |  | 17.62 | 5.51 | 1964.89 |
| SSP | Albania | El Porvenir (PO) | P41 | POT1P1 | RTV | 16.0 |  |  |  |  | 34 |  | 16.60 | 4.93 | 2117.20 |
|  |  |  | P42 | POT1P2 | RTT | 7.0 |  |  |  |  | 44 |  | 19.14 | 8.17 | 3049.23 |
|  |  |  | P43 | POT2P1 | RTV | 15.5 |  |  |  |  | 27 |  | 17.84 | 4.17 | 1781.04 |
|  |  |  | P44 | POT2P2 | RTT | 6.5 |  |  |  |  | 28 |  | 23.52 | 7.57 | 2233.17 |
|  |  |  | P45 | POT3P1 | RTV | 18.0 |  |  |  |  | 45 |  | 20.14 | 8.97 | 3199.93 |
|  |  |  | P46 | POT3P2 | PAD | 13.0 |  |  |  |  | 7 |  | 40.07 | 7.50 | 809.96 |
|  |  |  | P47 | POT4P1 | RTV | 17.0 |  |  |  |  | 36 |  | 23.34 | 9.25 | 2815.11 |
|  |  |  | P48 | POT4P2 | RTT | 10.0 |  |  |  |  | 82 | 3 | 20.97 | 8.75 | 6589.51 |
|  |  |  | P49 | POT5P1 | RTT | 6.0 |  |  |  |  | 39 |  | 23.69 | 9.59 | 3127.45 |
|  |  |  | P50 | POT5P2 | RTT | 6.0 |  |  |  |  | 32 |  | 23.20 | 8.77 | 2509.74 |
|  | El Doncello | La Vega (VE) | P51 | VET1P1 | RTV | 17.0 |  |  |  |  | 37 |  | 19.50 | 8.51 | 2566.18 |
|  |  |  | P52 | VET1P2 | RTT | 8.0 |  |  |  |  | 33 |  | 18.72 | 8.58 | 2271.65 |
|  |  |  | P53 | VET2P1 | RTT | 10.0 |  |  |  |  | 47 |  | 22.42 | 11.45 | 3619.62 |
|  |  |  | P54 | VET2P2 | BSE | 22.0 |  |  |  |  | 46 |  | 22.47 | 11.37 | 3540.80 |
|  |  |  | P55 | VET3P1 | RTV | 15.0 |  |  |  |  | 36 |  | 17.50 | 5.11 | 2331.78 |
|  |  |  | P56 | VET3P2 | BSE | 22.0 |  |  |  |  | 34 |  | 17.28 | 6.09 | 2206.27 |
|  |  |  | P57 | VET4P1 | RTT | 7.0 |  |  |  |  | 34 |  | 17.10 | 5.62 | 2197.94 |
|  |  |  | P58 | VET4P2 | RTV | 15.0 |  |  |  |  | 44 |  | 23.51 | 9.18 | 3445.97 |
|  |  |  | P59 | VET5P1 | RTT | 10.0 |  |  |  |  | 59 |  | 15.68 | 5.93 | 3597.80 |
|  |  |  | P60 | VET5P2 | RTT | 9.0 |  |  |  |  | 46 |  | 28.20 | 17.20 | 4107.41 |
|  | Milán | Esmeraldas (ES) | P61 | EST1P1 | RTT | 4.0 |  |  |  |  | 15 |  | 21.48 | 6.57 | 1100.43 |
|  |  |  | P62 | EST1P2 | RTT | 5.0 |  |  |  |  | 41 |  | 20.08 | 5.57 | 2942.94 |
|  |  |  | P63 | EST2P1 | RTT | 6.0 |  |  |  |  | 36 |  | 16.92 | 4.90 | 2296.48 |
|  |  |  | P64 | EST2P2 | RTT | 4.0 |  |  |  |  | 10 |  | 20.55 | 5.03 | 730.20 |
|  |  |  | P65 | EST3P1 | RTT | 6.0 |  |  |  |  | 22 |  | 18.51 | 5.67 | 1496.52 |
|  |  |  | P66 | EST3P2 | RTT | 4.0 |  |  |  |  | 10 |  | 18.84 | 5.88 | 686.38 |
|  |  |  | P67 | EST4P1 | PAD | 14.0 |  |  |  |  | 11 |  | 19.33 | 4.96 | 777.33 |
|  |  |  | P68 | EST4P2 | RTT | 7.0 |  |  |  |  | 43 |  | 13.73 | 5.13 | 2411.35 |
|  |  |  | P69 | EST5P1 | RTV | 18.0 |  |  |  |  | 53 |  | 26.13 | 9.62 | 4556.80 |
|  |  |  | P70 | EST5P2 | RTT | 8.0 |  |  |  |  | 49 |  | 21.10 | 7.97 | 3609.88 |
|  | Morelia | Villa Mery (VM) | P71 | VMT1P1 | RTT | 5.0 |  |  |  |  | 16 |  | 16.53 | 5.88 | 997.96 |
|  |  |  | P72 | VMT1P2 | RTT | 8.0 |  |  |  |  | 32 |  | 18.35 | 7.51 | 2150.62 |
|  |  |  | P73 | VMT2P1 | BSE | 20.0 |  |  |  |  | 18 |  | 15.02 | 9.79 | 1059.78 |
|  |  |  | P74 | VMT2P2 | BSE | 20.0 |  |  |  |  | 43 | 2 | 24.19 | 17.76 | 4139.71 |
|  |  |  | P75 | VMT3P1 | BSE | 20.0 |  |  |  |  | 40 | 3 | 21.36 | 13.23 | 4281.19 |
|  |  |  | P76 | VMT3P2 | BSE | 20.0 |  |  |  |  | 52 |  | 20.99 | 13.54 | 3801.80 |
|  |  |  | P77 | VMT4P1 | RTT | 4.5 |  |  |  |  | 30 |  | 13.88 | 8.08 | 1680.17 |
|  |  |  | P78 | VMT4P2 | RTT | 5.0 |  |  |  |  | 18 | 4 | 16.03 | 7.92 | 3371.90 |

VC: Vegetation covers; BSE: Secondary forest; CAC: Agroforestry cacao cultivation; PAD: Pastures with scattered trees; RTT: Early Fallows; RTV: Old Fallows. CS: Cacao shrubs; HbT: *Hevea brasiliensis* trees; CI: Citrus; FT: Fruit trees; ST: Stem trees; PA: Palms. Age: average in years. DBH: Diameter at breast height average in cm. Height tree: Average in m. AGB: Above-ground biomass (kg tree⁻¹).

**S3 Table. Results of the analysis of variance of total biomass and carbon stored in the different components in the eight landscape mosaics with agroforestry systems with cocoa and silvopastoral systems in the Colombian Amazon**. Values sharing a common letter in their means are not significantly different (p>0.05).

| **Mosaics** | **AGBt (Mg ha^-1^)** | | | **CAar (Mg ha^-1^)** | | | **CAhe (Mg ha^-1^)** | | | **CAho (Mg ha^-1^)** | | | **CAt (Mg ha^-1^)** | | |
| --- | --- | --- | --- | --- | --- | --- | --- | --- | --- | --- | --- | --- | --- | --- | --- |
|  | F = 0.65; p = 0.7147 | | | F = 1.47; p = 0.1909 | | | F = 11.92; p <0.0001 | | | F = 3.44; p = 0.0032 | | | F = 1.47; p = 0.1908 | | |
|  | **Mean** | **S.E.** |  | **Mean** | **S.E.** |  | **Mean** | **S.E.** |  | **Mean** | **S.E.** |  | **Mean** | **S.E.** |  |
| SR | 33.68 | 5.35 | ***A*** | 21.08 | 2.46 | ***A*** | 0.0011 | 0.0011 | ***BC*** | 0.0046 | 0.00078 | ***AB*** | 21.08 | 2.46 | ***A*** |
| VE | 28.72 | 5.86 | ***A*** | 17.32 | 2.46 | ***AB*** | 0.0060 | 0.0011 | ***C*** | 0.0049 | 0.00078 | ***AB*** | 17.32 | 2.46 | ***AB*** |
| PO | 30.17 | 5.86 | ***A*** | 16.35 | 2.46 | ***AB*** | 0.0016 | 0.0011 | ***BC*** | 0.0030 | 0.00078 | ***BC*** | 16.35 | 2.46 | ***AB*** |
| BA | 27.24 | 5.35 | ***A*** | 15.87 | 2.46 | ***AB*** | 0.0022 | 0.0011 | ***BC*** | 0.01 | 0.00078 | ***AB*** | 15.87 | 2.46 | ***AB*** |
| VM | 23.04 | 5.86 | ***A*** | 15.47 | 2.76 | ***AB*** | 0.0034 | 0.0012 | ***BC*** | 0.01 | 0.00087 | ***A*** | 15.57 | 2.76 | ***AB*** |
| TR | 19.81 | 6.55 | ***A*** | 13.85 | 2.46 | ***B*** | 0.0036 | 0.0011 | ***B*** | 0.0019 | 0.00078 | ***C*** | 13.85 | 2.46 | ***B*** |
| ES | 22.94 | 5.35 | ***A*** | 12.00 | 2.46 | ***B*** | 0.01 | 0.0011 | ***A*** | 0.01 | 0.00078 | ***A*** | 12.00 | 2.46 | ***B*** |
| TE | 23.38 | 5.35 | ***A*** | 11.97 | 2.46 | ***B*** | 0.0017 | 0.0011 | ***BC*** | 0.0046 | 0.00078 | ***AB*** | 11.97 | 2.46 | ***B*** |

SR: Santa Rosa; VE: La Vega ; PO: El Porvenir; BA: Batalla 13; VM: Villa Mery; TR: El Triunfo; ES: Esmeraldas; TE: El Tesoro; AGBt: Above-ground biomass; CAar: Carbon air; CAhe: Carbon herbaceus; CAho: Carbon Litter; CAt: Total Carbon. S.E: Standard Error.

**S4 Table. Results of the analysis of variance of total biomass and carbon stored in the different components in vegetation cover with partitions by agroforestry systems with cocoa and silvopastoral systems in the Colombian Amazon.** Values sharing a common letter in their means are not significantly different (p>0.05).

| **Variable** | **F-value** | **p-value** | **Vegetation covers** | | | | | | | | | | | | | | |
| --- | --- | --- | --- | --- | --- | --- | --- | --- | --- | --- | --- | --- | --- | --- | --- | --- | --- |
|  |  |  | **BSE** | | | **CAC** | | | **RTV** | | | **RTT** | | | **PAD** | | |
|  |  |  | **Mean** | **S.E.** |  | **Mean** | **S.E.** |  | **Mean** | **S.E.** |  | **Mean** | **S.E.** |  | **Mean** | **S.E.** |  |
| CAar | 3.71 | 0.0083 | 21.61 | 2.64 | ***A*** | 17.35 | 2.15 | ***AB*** | 17.31 | 1.81 | ***AB*** | 13.87 | 1.26 | ***BC*** | 8.02 | 3.04 | ***C*** |
| CAhe | 3.87 | 0.0066 | 0.00087 | 0.0016 | ***B*** | 0.0020 | 0.0013 | ***AB*** | 0.00094 | 0.0011 | ***B*** | 0.0048 | 0.00074 | ***A*** | 0.01 | 0.0018 | ***A*** |
| CAho | 3.30 | 0.0152 | 0.01 | 0.00091 | ***A*** | 0.0036 | 0.00075 | ***AB*** | 0.01 | 0.00063 | ***A*** | 0.0048 | 0.00044 | ***A*** | 0.0015 | 0.0011 | ***B*** |
| CAt | 3.71 | 0.0083 | 21.61 | 2.64 | ***A*** | 17.35 | 2.15 | ***AB*** | 17.31 | 1.81 | ***AB*** | 13.87 | 1.26 | ***BC*** | 8.02 | 3.04 | ***C*** |
| **SAFc** | | | | | | | | | | | | | | | | | |
| CAar | 3.85 | 0.0108 | 31.45 | 5.41 | ***A*** | 17.35 | 2.21 | ***B*** | 18.00 | 2.55 | ***B*** | 11.99 | 2.12 | ***B*** | 9.66 | 3.83 | ***B*** |
| CAhe | 1.84 | 0.1437 | 0.00 | 0.0020 | ***A*** | 0.0020 | 0.00080 | ***A*** | 0.00056 | 0.00093 | ***A*** | 0.0033 | 0.00077 | ***A*** | 0.0035 | 0.0014 | ***A*** |
| CAho | 1.19 | 0.3308 | 0.01 | 0.0018 | ***A*** | 0.0036 | 0.00072 | ***A*** | 0.01 | 0.00083 | ***A*** | 0.0042 | 0.00069 | ***A*** | 0.0023 | 0.0013 | ***A*** |
| CAt | 3.85 | 0.0108 | 31.45 | 5.41 | ***A*** | 17.35 | 2.21 | ***B*** | 18.00 | 2.55 | ***B*** | 11.99 | 2.12 | ***B*** | 9.66 | 3.83 | ***B*** |
| **SSP** | | | | | | | | | | | | | | | | | |
| CAar | 2.03 | 0.1284 | 18.34 | 2.83 | ***A*** |  |  |  | 16.54 | 2.45 | ***A*** | 14.98 | 1.48 | ***AB*** | 4.75 | 4.90 | ***B*** |
| CAhe | 3.33 | 0.0308 | 0.0012 | 0.0022 | ***B*** |  |  |  | 0.0014 | 0.0019 | ***B*** | 0.01 | 0.0011 | ***AB*** | 0.01 | 0.0038 | ***A*** |
| CAho | 2.64 | 0.0651 | 0.01 | 0.0011 | ***A*** |  |  |  | 0.01 | 0.00096 | ***A*** | 0.01 | 0.00058 | ***A*** | 0.00 | 0.0019 | ***B*** |
| CAt | 2.03 | 0.1283 | 18.34 | 2.83 | ***A*** |  |  |  | 16.54 | 2.45 | ***A*** | 14.98 | 1.48 | ***AB*** | 4.75 | 4.90 | ***B*** |

CAar: Carbon air (Mg ha^-1^); CAhe: Carbon herbaceus (Mg ha^-1^); CAho: Carbon Litter (Mg ha^-1^); CAt: Total Carbon (Mg ha^-1^). BSE: Secondary forest; CAC: Agroforestry cacao cultivation; PAD: Pastures with scattered trees; RTT: Early Fallows; RTV: Old Fallows. S.E: Standard Error.
