# Supplementary material for: Influence of landscape structure on carbon storage in agroforestry systems with cacao and silvopastoral systems in the Colombian Amazon
Source: PLoS One. 2025 Jun 23;20(6):e0325477. doi: 10.1371/journal.pone.0325477 (PMC12184915; doi:10.1371/journal.pone.0325477)
Supplement: Attachment — Submitted filename: Response to Reviewers.pdf [file pone.0325477.s004.pdf]

Florencia, Colombia, April 14, 2025

Dra.
Marcela Pagano, Ph.D, M.D.
Academic Editor
PLOS ONE

Greetings,

Respectfully, the authors of the manuscript titled "**Influence of Landscape Structure on Carbon Storage in Agroforestry Systems with Cacao and Silvopastoral Systems in the Colombian Amazon**" hereby submit the "**Response to Reviewers**" letter for your consideration. In this document, we provide detailed responses to each of the questions, suggestions, and comments raised during the review process. o facilitate the understanding of this document, we would like to inform that the responses to each question are provided at the end of each section, highlighted in bold text. We trust that we have been thorough and detailed in addressing the queries.

Reviewer #1

In the manuscript, the authors did not include much data and objective in the abstract. **Response: The abstract has been revised to explicitly include the overarching objective of the study. Furthermore, additional data have been incorporated into the manuscript to enhance the robustness and clarity of the presented results.**

There is also a need to incorporate the objective in the introduction section. **Response: we have included the overall objective of the study at the end of the Introduction section to provide greater clarity regarding the scope and intent of the research.**

Please write the aim and applicability of the study. **Response: The overall objective of the research has now been explicitly stated in both the Abstract and the Introduction sections to enhance clarity and align with the manuscript's scientific narrative.**

There is a need to include study site photographs for authentication and to clarify the hypothesis of this study. **Response: Figure 2 has been incorporated to include representative photographs of the different types of vegetation cover identified within the eight landscape mosaics characterized by cacao agroforestry systems (AFS) and silvopastoral systems (SPS) studied. These images aim to enhance the visual understanding of the structural heterogeneity and composition of the land cover types across the sampled landscapes.**

Please add many more recent references related to the study. **Response: We conducted a more in-depth literature review to identify studies as closely related as possible to the objectives of our research. As a result, we have included additional references and incorporated further biological explanations to support the interpretation of our findings. It is important to clarify that, for our study area—the Colombian Amazon—this work represents a pioneering effort in analyzing, at a landscape scale, the causal relationships between landscape structure and configuration and an ecosystem service of high relevance: carbon sequestration. This is particularly significant in the context of climate change mitigation initiatives.**

I strongly suggest that the author edit and rewrite the sentences for language editing, which is the most useful criterion of any scholarly research article. **Response: A thorough revision of the manuscript was conducted, with particular attention to improving the overall writing style. This effort aimed to ensure a high level of coherence and clarity in the presentation of ideas throughout the text.**

Discussion of the manuscript needs to elaborate by including some recent literature. **Response: In collaboration among the three authors, the presentation of the results has been restructured. This revision allowed for a more comprehensive analysis and an expanded discussion, now supported by a broader range of relevant literature and recent studies.**

Reviewer #2

I reviewed the manuscript titled "Influence of Landscape Structure on Carbon Storage in Agroforestry Systems with Cacao and Silvopastoral Systems in the Colombian Amazon" for consideration in PLOS ONE. This study addresses a critical issue in the conservation of Amazonian ecosystems by evaluating the influence of landscape structure and configuration on carbon storage in cacao agroforestry systems (SAFc) and silvopastoral systems (SSP). The study provides valuable insights into the role of agroforestry mosaics in carbon regulation, highlighting their potential for climate change mitigation. The research suggests strategies to enhance connectivity and increase carbon sequestration capacity in productive landscapes, which has significant implications for land-use planning. However, I do have some suggestions and areas for improvement for the manuscript:

• Biomass and carbon storage may vary depending on the floristic composition within SAFc and SSP, suggesting the need for further studies on the contribution of key species. **Response: As noted in the methodology section, we included a general description of the composition and structure of the sampled cacao-based agroforestry systems and silvopastoral systems in the Colombian Amazon. This characterization aimed to provide essential context for interpreting the ecological dynamics and functional patterns observed across the studied land-use systems.**

• Differences in management practices within agroforestry systems could affect carbon dynamics, which could be explored in greater depth. **Response: In the description of the systems within the study area, we included a detailed account of the management characteristics specific to each system. This information was incorporated to provide a comprehensive understanding of the context in which each system operates, thereby supporting the interpretation of the results within their respective socio-ecological frameworks.**

• The statistical analysis and results section presents valuable insights, but there are several areas where clarity, coherence, and interpretation could be improved. The results section presents p-values and F-statistics but lacks biological or ecological interpretation of the findings. I suggest adding means and SD of each variable to give a better perspective of the values and differences and explain the ecological significance of significant and non-significant results. **Response: Both the description of the statistical analysis section and the presentation of the results have been revised to include the means, standard deviations, and standard errors for each analyzed variable. Additionally, the supplementary materials file has been consolidated to present the**

results of the conducted analyses, as well as the matrix of the field-measured dendrometric variables.

• Add a brief explanation of why each statistical method was chosen and how it aligns with the study objectives. **Response: The necessary adjustments have been made to the statistical analysis section of the manuscript in accordance with the feedback provided.**

• The manuscript does not mention whether key assumptions for ANOVA (normality, homogeneity of variance) and regression analyses (linearity, multicollinearity) were checked. **Response: The statistical analysis section of the manuscript has been reorganized and adjusted in accordance with the reviewers' suggestions. We believe these modifications enhance the clarity and rigor of the analyses presented.**

• Some tables and figures lack clear captions or explanations, making it difficult for readers to interpret the data. Please improve the clearly label significant results with asterisks or superscripts in tables. Ensure figure legends describe all abbreviations and variables. For example, table 3 presents mean carbon storage values across different vegetation covers. Add a legend indicating that values with the same letter are not significantly different (Tukey's HSD test, $p > 0.05$). **Response: We have made the necessary adjustments to the statistical analyses in the corresponding section. Additionally, we refined the presentation of the results. Several figures that were previously unclear in conveying the results have been modified, and their descriptions have been elaborated in the figure legends to enhance the clarity and interpretation of the data presented.**

• The results do not mention potential confounding factors that could influence carbon storage variability (e.g., soil type, elevation, past management). Please address potential confounders and, if applicable, describe how they were controlled. **Response: In the expanded and revised discussion of the results, we have addressed potential factors that may influence carbon storage. This revision includes a comprehensive analysis of the underlying variables, incorporating both biotic and abiotic elements that could significantly affect carbon sequestration in the studied system.**

• The PCA and PLS regression results are presented with numerical outputs do not have a clear biological interpretation. Please explain what the principal components represent and how the regression findings contribute to the study's conclusions. **Response: We have taken into account the potential confusion arising from the presentation of both PCA and PLS regression analyses. Consequently, we have prioritized the inclusion of the PLS triplot, as it effectively combines the features of Principal Component Analysis (PCA) and Multiple Regression, offering a more comprehensive representation of the data. We believe this approach enhances clarity and provides a more integrated view of the results.**

• The discussion section provides a general overview of the findings, but it would benefit from a more in-depth interpretation of the observed patterns. **Response: In response to the valuable and insightful recommendations provided by the reviewers, a comprehensive literature search was conducted, and the analysis of the results presented in the manuscript has been significantly strengthened. We have carefully incorporated the latest relevant studies to ensure a robust and thorough discussion of the findings. The revisions aim to enhance the clarity and depth of the analysis, aligning it with current advancements in the field.**

• I suggest explicitly addressing how different landscape metrics influence carbon sequestration and why certain patch configurations lead to higher/lower storage. **Response: In line with the central objective of this study, we clearly addressed the relationship between carbon storage components, landscape mosaics, and the vegetative covers classified within the agroforestry systems involving cacao and silvopastoral systems. We also examined the metrics that define the structure and spatial configuration of the studied landscapes.**

• The discussion includes citations to previous studies but lacks a critical comparison that highlights the novelty of the current research. Clearly articulate how the results confirm, contradict, or expand on prior studies. **Response: The authors believe that this recommendation has been adequately addressed. A coherent explanation of the results was provided, with a thorough comparison to previous studies related to both the subject and the object of investigation. Furthermore, these results were contrasted with studies conducted in the specific region of interest, ensuring a comprehensive context for the findings.**

• Although different landscape mosaics are analyzed, microclimatic and edaphic factors that may influence carbon capture are not explored in detail. **Response: In the adjusted results discussion, we included an analysis of the environmental and climatic variables of the region that are influencing, and may also be affected by, changes in the spatial structure and configuration of the mosaics with SAF and SSP.**

• The discussion would benefit from explicitly acknowledging limitations and suggesting future research directions. **Response: In the discussion, we have included several general recommendations regarding landscape management, emphasizing the importance of maintaining structural connectivity and addressing fragmentation issues. These measures are aimed at enhancing the provision and/or maintaining the regulation of ecosystem services, such as carbon storage, which is critical for sustaining ecological functions and mitigating climate change.**

• The discussion highlights the importance of landscape connectivity but does not explicitly state how land-use policies should integrate these findings. Please expand the implications by providing concrete recommendations for conservation planning. **Response: The manuscript includes considerations regarding the management and governance of rural landscapes, particularly focusing on the spatial structure and configuration of landscapes within agroforestry systems involving cacao and silvopastoral systems in the Amazon region. These aspects are critical for understanding how the integration of such systems can influence both ecological dynamics and sustainable land use practices in this unique and vital biome.**

Reviewer #3:

The manuscript deals with an interesting subject involving landscape metrics and measurements of biomass and carbon in vegetation in the field. The authors indicate that this type of study is a pioneer in the region. However, I found the article difficult to understand for a general audience, and it had a weak connection between the objectives, the methodology, and the results. I have tried to highlight some of the critical points below. A conceptual issue that seems important to me is the classification of silvopastoral systems, which involve trees, pastures, and animals. The system closest to the silvopastoral system (PAD) is poorly represented. So, it does not seem easy

to relate landscape metrics to the carbon stored in production systems (SAF; SPP). In addition, some results are conflicting, such as the carbon in the aerial biomass of grasses being higher than in secondary tropical forests; estimation of carbon stored in the biomass of trees, herbaceous, and litter in LAG=lagoons; use of biomass equations for fruit species, but they are not highlighted in any production system. In addition, the manuscript needs to reorganize the text and its writing. I could not recommend the acceptance of the manuscript in its present form. **Response: We sincerely appreciate your thoughtful comment, which has greatly contributed to the substantial improvements in both the presentation of our results and the analysis provided. We believe that the revisions made address your concerns effectively and that the manuscript now meets the scientific rigor and the submission guidelines outlined for authors. We are confident that these adjustments make the paper suitable for publication in the journal.**

Material and methods

The separation of the M&M item could be redone. Line 148 onwards should not be part of the study area. **Response: We have revised the manuscript to include a new section that distinctly outlines the methods used for analyzing the landscape structure and configuration in the study area. This additional section specifically describes the analysis of agroforestry systems involving cacao and silvopastoral systems, which were the focus of the study.**

L148-149. Could it include the main species that make up the SSP? Does SAF with cocoa present high or low plant diversity? **Response: In response to the new revisions and adjustments, we have included a comprehensive description in the Methods section detailing the composition and structure of the agroforestry systems with cacao and the silvopastoral systems sampled in the Colombian Amazon. This description now highlights the key species that constitute each system, providing a clearer understanding of their ecological and functional characteristics.**

L170-173. What would be the area of each of the 78 plots? How were the plots within each mosaic systematized? **Response: The area of each plot is presented in the abstract of the initial manuscript. In response to the reviewer's comment, we have added clarification in the Methods section, specifically in the part describing the quantification of biomass in carbon deposits. It is now explicitly stated that each plot covers an area of 0.1 hectares, with dimensions of 20x50 meters. We have also included the relevant citation that suggests the appropriate plot size.**

L99-211. This topic is confusing. How was the carbon stored in each plot converted to SAF, SPP, and mosaic model scale? Did the authors use the same equation to convert aerial biomass into root biomass for all vegetation types? **Response: In the "Statistical Analysis" section, we have expanded the explanation regarding the extrapolation of carbon storage from individual plots to the broader vegetation cover areas, as well as to the mosaic areas and the two studied systems. To estimate root biomass, we employed the indirect method using the model proposed by Cairns et al. (1997) and the IPCC (2003) guidelines, which are specifically recommended for tropical areas. This approach was applied across all vegetation types in the study.**

Why were landscape metrics classified as descriptive to use in PCA if they are quantitative in regression? **Response: Landscape metrics are inherently quantitative in nature; however, they were used in a descriptive manner within the Principal Component Analysis (PCA) because the primary aim of this analysis was to reduce dimensionality and explore general patterns in landscape structure without assuming causal relationships. In this context, these metrics were employed as explanatory variables to summarize landscape variability, rather than to make direct statistical inferences. Nevertheless, in response to the reviewer's recommendations, we have decided to omit this analysis in the revised manuscript, as we now utilize the same metrics within the Partial Least Squares (PLS) framework.**

Moreover, why do linear regression and PLS? **Response: Linear regression (LR) was employed to establish a direct relationship between a landscape metric and the response variable, under the assumptions of independence and low collinearity. However, partial least squares regression (PLS) was used in cases of high collinearity among the explanatory variables (landscape metrics). PLS addresses this issue by constructing latent components that maximize covariance with the response variable, proving more robust in contexts with highly correlated variables and relatively small sample sizes. In this regard, we have decided to remove the linear regressions from the manuscript, as the relationships between the variables resulted in R^2 values not exceeding 0.3.**

Are the authors using the same metrics as Table 1 for all cases (PCA, LR, SLS)? **Response: Initially, yes, the same set of metrics described in Table 1 was used. However, the final selection of variables may vary depending on the specific analysis performed. In the case of PCA, all available metrics were included in order to identify the axes of greatest variation. For linear regression and PLS, variable selection was based on correlation, multicollinearity, or predictive importance. Therefore, some metrics may have been excluded if they did not contribute relevant information or if their inclusion led to statistical issues.**

How did the authors deal with the different measurement scales to do the PCA? **Response: In order to prevent variables with larger scales from dominating the results of the Principal Component Analysis (PCA), all metrics were standardized (transformed to a mean of zero and a standard deviation of one) prior to the analysis. This approach is commonly employed in PCA when variables differ in units or magnitudes, ensuring that each variable contributes equally to the analysis.**

In general, PCA can be influenced by high and low values. **Response: Yes, this is correct. To mitigate this effect, a preliminary inspection for outliers in the landscape metrics was conducted, and extreme values that could compromise the stability of the analysis were excluded. This approach ensured that the PCA captured the general patterns rather than anomalous cases.**

Results

A more coherent separation of the results into topics would facilitate understanding.
It is unclear what type of ecosystem fits the silvopastoral systems (SPP) classification. The only one that could fit is the PAD model, represented only by the ES Mosaic. So, I asked how it was possible to statistically test the effects of the SAF and SPP models on the stored carbon.

Response: We have restructured the presentation of the results section in the revised manuscript to enhance clarity and facilitate interpretation. Specifically, the results are now organized into three main components: (1) landscape structure and configuration metrics, (2) carbon stocks across the different studied components, and (3) the relationships between landscape metrics and carbon storage across mosaics and vegetation cover types. This reorganization aims to improve the logical flow of the findings and strengthen the link between landscape patterns and ecosystem carbon dynamics.

Also, how did the authors calculate the carbon stored in trees in LAG, RIO, PEN, PPH, PPL, RTT, and RTV? **Response: We have revised the classification of vegetation cover types where the plots for biomass and carbon inventories were established. As described in the methodology, carbon stocks were estimated using a carbon fraction of 0.47 applied to the total aboveground biomass. This biomass was calculated using the allometric models detailed in Table 2. As shown in Table S2 of the supplementary material, a complete census was conducted for all trees with a diameter at breast height (DBH) greater than 10 cm. In the case of cacao shrubs, stem diameter was measured at 30 cm above ground level (D30), as appropriate for their growth form. These measurements were taken within each plot, after which the corresponding allometric model was applied to estimate biomass.**

Table 3. The table is cut off, and I cannot see all the information. Furthermore, what is the unit of values? Is the size of the area in ha, m2, km2? Why isn't there SD in some of the information? Why are the letters that indicate statistical results only in CAC and PPL? **Response: Given the complexity of Table 3, it has been thoroughly revised. The vegetation cover classes were reclassified accordingly, and both the mean area and standard deviation for each class were included, expressed in hectares. The table caption was updated to clearly reflect these changes. Additionally, statistical grouping letters were assigned to all vegetation cover types to indicate significant differences**.

L230-245. The authors indicate the different landscape metrics in the PCA, but it would be easier to relate them to Table 1. For example, which metrics are related to "complexity in patch shape, while the negative end associates with mosaics featuring larger, more aggregated patches" "mosaics with irregular, aggregated patches, while the negative end is characterized by mosaics with larger patches that have smoother, less irregular contours". **Response: In response to this recommendation, we have included Table 1, which details the landscape metrics analyzed in this study. The table is structured in three columns: the first column describes the specific landscape property being evaluated—namely, composition, patch aggregation within the mosaic, and spatial configuration, which refers to the shape and complexity of both individual patches and the overall mosaic. The second column provides the abbreviations used throughout the manuscript for each metric. The third column offers a concise explanation of each metric, clarifying what it evaluates and what it indicates in the context of landscape structure.**

L255-264. This paragraph should be in the M&M. In addition, the authors could go into more detail about this type of information:
"The cover type with the highest number of plots was early fallow (RTT), followed by old fallow (RTV) and agroforestry cacao cultivation (CAC). A total of 2,846 individual plants were surveyed across both systems: 1,505 trees in the SAFc mosaics, including 330 cacao shrubs, 105 rubber trees (H. brasiliensis), 29 palms, thirteen fruit trees, and 1,028 saplings." **Response: In**

accordance with the reviewer's suggestion, the paragraph has been integrated into the Methods section to enhance clarity and methodological transparency.

L266-276. Given the diversity of ecosystems and the very different number of replicates, comparing the SAF and SPP systems seems difficult. In addition, it seems strange to me that the biomass of grasses is greater than forest (BSE). The carbon stored in the soil could be higher in grass than in secondary forest, but I had not yet seen biomass. **Response: Considering the diversity of vegetation cover types, we performed a reclassification to improve the consistency and representation of replicate plots across cover categories. This adjustment allowed for a more balanced comparison and increased statistical robustness. As a result, secondary forests exhibited the highest aboveground biomass and carbon stocks, whereas pasturelands with scattered trees showed the highest herbaceous biomass and associated carbon. It is important to note that this study did not include estimates of soil carbon storage.**

Fig.3. The figure does not show ANOVA, but rather the average carbon values in biomass. **Response: The figure has been removed and replaced with Table 4, which presents the results of the analysis of variance using generalized linear mixed models. This table includes mean values, standard errors, and significance groupings based on p-values for carbon content across all studied components (aboveground biomass, herbaceous layer, and litter). Additionally, this information is provided in greater detail in the Supplementary Material.**

A period is used and not a comma to separate the numbers in English. **Response: the issue was an unintentional error stemming from the original manuscript, which was written in Spanish. The translation has now been corrected, and the entire text has been carefully reviewed to ensure consistency and accuracy throughout.**

L289. How did the authors arrive at this value of stored carbon? **Response: The Statistical Analysis section provides a detailed description of the method used to estimate carbon fraction based on biomass data, as well as the procedures for extrapolating these estimates to the area of the vegetation cover types and mosaic landscapes. As specified in the manuscript, the extrapolated area encompasses 800 hectares across the two productive systems evaluated within the study area.**

L305-303. Is carbon stored in biomass equal to total carbon? Usually, grasses have a lot of root biomass. **Response: We agree with the reviewer's observation. The carbon stored in aboveground biomass is indeed distinct from that stored in herbaceous vegetation and litter. As shown in Table S4 of the Supplementary Material, pastures with scattered trees—typically dominated by grasses—exhibit higher levels of carbon stored in herbaceous vegetation. This distinction underscores the structural and functional differences among vegetation strata, which contribute differentially to carbon pools within these silvopastoral systems.**

Discussion

L419. This connectivity metric is unclear. Where is this result? **Response: Table 1 presents the metrics associated with the aggregation properties of patches of the same class within the mosaic, along with the results obtained. These results are further detailed in Tables and**

Figures 3 through 5, where the dispersion and aggregation levels of the patches are shown. Additionally, these figures provide an explanation of the connectivity across the different mosaics.

L 428-432. This relationship was weak many times for some metrics. Could you explain which metrics might be most important for estimating carbon relative to Table 1? Although the linear regression was significant, the data distribution in Fig 4 does not appear linear. **Response: With the adjustments made, we were able to analyze that the most important common metrics for landscapes with both cacao agroforestry systems and silvopastoral systems are related to aggregation. The patch size and shape metrics within the mosaics were found to be differently associated with variation in carbon content across the components.**

In response to the valuable comments, suggestions, and recommendations provided by the reviewers, the authors would like to inform that all feedback has been thoroughly addressed with careful attention to detail. This process was undertaken with the goal of ensuring the manuscript aligns with the high scientific standards expected by this journal, presenting a coherent, well-structured narrative that effectively contributes to the field.

We sincerely appreciate your attention to this matter.

Jenniffer Tatiana Díaz-Cháux
University of the Amazon
Florencia, Colombia

Alexander Velasquez-Valencia
University of the Amazon
Florencia, Colombia

Fernando Casanoves
CATIE
Turrialba, Costa Rica
